# Real-world observations and impacts of Chinese herbal medicine for migraine: results of a registry-based cohort study

**DOI:** 10.3389/fphar.2024.1330589

**Published:** 2024-02-02

**Authors:** Shaohua Lyu, Claire Shuiqing Zhang, Anthony Lin Zhang, Xinfeng Guo, Rong Hua, Zhenhui Mao, Qiaozhen Su, Charlie Changli Xue, Jingbo Sun

**Affiliations:** ^1^ The Second Affiliated Hospital of Guangzhou University of Chinese Medicine, Guangdong Provincial Hospital of Chinese Medicine and Guangdong Provincial Academy of Chinese Medical Sciences, Guangzhou, China; ^2^ The China-Australia International Research Centre for Chinese Medicine, STEM College, RMIT University, Melbourne, VIC, Australia; ^3^ State Key Laboratory of Dampness Syndrome of Chinese Medicine, Guangzhou, China; ^4^ Guangdong Provincial Key Laboratory of Research on Emergency in TCM, Guangzhou, China

**Keywords:** migraine, Chinese herbal medicine, real-world, cohort study, preferences and values, clinical expertise

## Abstract

**Background:** Migraine is a prevalent, recurrent condition with substantial disease burden. Chinese herbal medicine (CHM) has been used frequently for migraine in controlled clinical settings. This study is to summarise the characteristics of patients who seek clinical care in a tertiary Chinese medicine hospital in China; to gather their preferences and values of using CHM; to explore the effect of CHM for migraine and its comorbidities in a real-world setting, and to collect first-hand expertise of clinicians’ practice pattern in prescribing CHM for migraine.

**Methods:** This registry-based cohort study was prospectively conducted at Guangdong Provincial Hospital of Chinese Medicine from December 2020 to May 2022. Adult migraine patients seeking their initial anti-migraine clinical care at the hospital were consecutively recruited and followed up for 12 weeks. Practitioners specialised in headache management prescribed individualised treatments without research interference. Standardised case report forms were employed to gather information on patients’ preferences and perspective of seeking clinical care, as well as to assess participants’ migraine severity, comorbidities, and quality of life, at 4-weeks intervals. Various analytical methods were utilised based on the computed data.

**Results:** In this study, we observed 248 participants. Of these, 73 received CHM treatment for 28 days or longer. Notably, these participants exhibited a greater disease severity, compared to those treated with CHM for less than 28 days. Of the 248 participants, 83.47% of them expected CHM would effectively reduce the severity of their migraine, around 50% expected effects for migraine-associated comorbidities, while 51.61% expressing concerns about potential side effects. CHM appeared to be effective in reducing monthly migraine days and pain intensity, improving patients’ quality of life, and potentially reducing comorbid anxiety, with a minimum of 28 days CHM treatment. Herbs such as *gan cao*, *gui zhi*, *chuan xiong*, *fu ling*, *bai zhu*, *yan hu suo*, etc. were frequently prescribed for migraine, based on patients’ specific symptoms.

**Conclusion:** CHM appeared to be beneficial for migraine and comorbid anxiety in real-world clinical practice when used continuously for 28 days or more.

**Clinical Trial Registration:** clinicaltrials.gov, identifier ChiCTR2000041003.

## 1 Introduction

Migraine is a primary headache disorder characterised by recurrent, unilateral, pulsing or throbbing, moderate to severe headaches (Headache Classification Committee of the International Headache Society IHS, 2018). It is prevalent among 14% of global population (Stovner et al., 2022), and ranked as the second disabling condition with 42.1 million of global age-standardised years lived with disability (YLDs) (Safiri et al., 2022). Notably, females are more susceptible to migraine, and tend to report heightened migraine severity and associated disability (Pavlovic et al., 2017; Vetvik and MacGregor, 2017; Lipton et al., 2018). Additionally, migraine commonly coexists with anxiety, depression and insomnia (Kelman and Rains, 2005; Freedom and Evans, 2013; Buse et al., 2020; Caponnetto et al., 2021), and these comorbidities, in return, exacerbate the burden of migraine (Kelman, 2007; Walters et al., 2014; Seng et al., 2017; Buse et al., 2020; Klonowski et al., 2022) and predict a less favourable prognosis (Bigal and Lipton, 2006; Lipton et al., 2019a).

Migraine is conventionally managed by prophylactic medications to reduce the frequency and severity of migraine attacks, as well as acute medications for temporary relief of pain and associated symptoms (Evers et al., 2009; Pringsheim et al., 2012; Worthington et al., 2013; Orr et al., 2015; Scottish Intercollegiate Guidelines Network SIGN, 2018; Kouremenos et al., 2019; Kowacs et al., 2019; Ailani et al., 2021; Diener et al., 2022; Domitrz et al., 2022; Dong et al., 2022; Wu et al., 2022; The British Association for the study of headache BASH, 2023). However, lack of efficacy and undesirable side effects associated with the conventional pharmacotherapies were widely reported (Malik et al., 2006; Blumenfeld et al., 2013; Ford et al., 2017; Lipton et al., 2019b; Takeshima et al., 2019; Ueda et al., 2019; Lombard et al., 2020; Hirata et al., 2021; Kim et al., 2021). Inadequate treatment responses can lead to increased reliance on acute medications, while overuse of acute medications has emerged as a significant risk factor for migraine chronification (Xu et al., 2020). Effective patient education can potentially reverse the overuse of acute medications (Probyn et al., 2017). Investigation on patients’ preferences and values, especially the knowledge and behaviour regarding acute medication use, could form the basis for developing a customised patient education strategy.

Furthermore, due to the limitations of pharmacotherapies, migraine patients often seek complementary and alternative treatments, including Chinese herbal medicine (CHM), to complement their current treatment strategies (Wells et al., 2011; Rhee and Harris, 2018). In China, CHM is prescribed to over 60% of outpatient migraine cases according to a retrospective analysis of the China Health Insurance Research Association medical insurance claims database (Yu et al., 2020). Meta-analyses of randomised controlled trials (RCTs) have demonstrated the effectiveness of CHM for migraine in controlled settings (Zhou et al., 2013; Li et al., 2015; Lyu et al., 2020; Lyu et al., 2022a). However, this existing evidence has limitations in terms of generalisability because of the highly selective eligibility criteria, unified interventions, and predefined treatment duration in RCT designs. CHM therapies in RCTs with these constraints do not align with real-world Chinese medicine clinical practices. As revealed by our earlier real-world analysis based on medical records, migraine patients with varied comorbidities received individually tailored CHM prescriptions over varying treatment durations (Lyu et al., 2022b). Quantitative evaluation is needed to assess the real-world effects of CHM on migraine and its comorbidities, complementing the evidence from RCTs. Moreover, the distinct patient profiles encountered in real-world clinical practice, along with their preferences and values regarding treatments from a Chinese medicine hospital, have been insufficiently explored. Nonetheless, this information has the potential to provide valuable insights for informed medical decision-making in clinical practice.

In light of these considerations, a prospective registry-based cohort study was undertaken to bridge the gap between research evidence and real-world clinical practice, and to support evidence-based Chinese medicine practice in managing migraines (Lyu et al., 2022c). The present manuscript is to portray a real-world representation of patients’ clinical characteristics, their preferences and values, their utilisation of treatment, and their responses to CHM interventions within the context of the studied Chinese medicine hospital.

## 2 Methods

### 2.1 Study design

This registry-based cohort study was undertaken at the Headache Department of the Guangdong Provincial Hospital of Chinese Medicine (GPHCM), a tertiary hospital in southern China (Guangdong Provincial Hospital of Chinese Medicine, 2021). Participant recruitment and follow-up observations commenced in December 2020 and ended in May 2022.

The study was approved by the ethics committee of GPHCM (ZE 2020-243-01) and registered with the Human Research Ethics Committee of RMIT University (#24235). Conduction of the study complied with the Declaration of Helsinki, Ethical Guidelines for Medical Research on Humans (World Medical Association, 2013), and the reporting of this study abides by the Strengthening the Reporting of Observational Studies in Epidemiology (STROBE) statement for cohort studies (von Elm et al., 2007).

#### 2.1.1 Eligibility criteria

Adult migraine patients seeking anti-migraine treatments in the studied Chinese medicine hospital for the first time were eligible for the study. Once patients were confirmed with a diagnosis of migraine according to the International Classification of Headache Disorders, third edition (ICHD-3) (Headache Classification Committee of the International Headache Society IHS, 2018), and were prescribed tailored treatments by headache specialists, they were invited to provide a written informed consent of participating in the study. A consecutive sampling method was applied to screen and recruit participants, as it is the best nonprobability sampling methods at controlling sampling bias (Polit and Beck, 2020). Migraine patients would be excluded for registration and participation if they were not capable of giving written informed consent or completing case report forms (CRFs).

#### 2.1.2 Intervention

Headache specialists prescribed individually tailored treatments to each participant, without additional interference from our research team. Participants would decide their treatment duration, with guidance from their headache specialists. Treatment details were later collected from participants’ medical records at completion of the 12-week observation period.

#### 2.1.3 Data collection

Case report form was utilised to collect data. The CRFs encompassed a set of standardised questions and several validated questionnaires, to collect the demographic and general information, patients’ preferences and values, migraine severity (including monthly migraine frequency, monthly migraine days, peak pain measured by numeric rating scale (NRS) and migraine duration), migraine comorbidities (anxiety assessed by generalised anxiety disorder 7-item (GAD-7), depression by patient health questionnaire-9 (PHQ-9) and insomnia by insomnia severity index (ISI)), and migraine-specific quality of life (MSQ, in domains of role function-restrictive (RFR), role function-preventive (RFP), and emotional function (EF)). More details can be referred to the published study protocol and, the Chinese Clinical Trial Registry (No. ChiCTR2000041003) (Lyu et al., 2022c).

Participants completed their CRFs with assistance of researchers during their initial evaluation, via either hardcopy or digital web link. Subsequent rounds of data collection occurred at week 4, week 8 and week 12. Scheduled reminders were sent to participants to enhance their compliance during the follow-up period. Participants were encouraged to maintain a digital migraine diary to document their migraine attacks throughout the observation period, which was then cross-referenced with data collected from their CRFs.

#### 2.1.4 Exposure and confounders

In this real-world cohort study, CHM treatment was predefined as the main exposure factor, and exposure levels were further measured by duration of CHM treatment. The participants were divided into two subgroups based on a cut-off duration of CHM treatment at 28 days, which is recommended as the least duration for migraine prophylaxis by clinical guidelines (Scottish Intercollegiate Guidelines Network SIGN, 2018; Dong et al., 2022):

• Subgroup A: CHM ≥28 days
• Subgroup B: CHM <28 days

In addition, comorbidities of migraine, along with gender, baseline severity of migraine, and aura, were predefined as confounders.

#### 2.1.5 Number of participants

This registry-based cohort study aimed to gather and investigate the real-world data in terms of migraine management and was conducted within a specific timeframe using a migraine cohort from a tertiary hospital. Given that no pre-determined hypotheses were to be tested, the necessity for sample size calculation was obviated (Gliklich et al., 2014).

### 2.2 Analytical methods

Data analyses were performed based on imputed dataset, which was dealt with ‘multiple imputation’ by SPSS in advance (IBM Corp, 2017). Continuous variables such as monthly migraine days were described with mean values and standard deviation, and compared between subgroups using *t*-test. While categorical variables, like gender, were presented as frequencies and percentages, and compared using chi square test. In addition, generalised linear mixed models (GLMM) were utilised to analyse repeated-measured outcomes. The GLMMs accommodate both normally and non-normally distributed dependent variables, allowing for the incorporation of covariates and factors. GLMMs with random effects were deemed appropriate to analyse repeated-measured, and longitudinal data from the same subjects (Cnaan et al., 1997; IBM, 2021). During conduction of GLMM analyses, continuous variables such as age, disease duration and the corresponding baseline assessment of the dependent variable, dichotomous variable of chronic migraine, and time-varying dichotomous variables of western medication usage for migraine prophylaxis and acute pain relief, were included as covariates.

## 3 Results

### 3.1 Summary of the study

**FIGURE 1:**
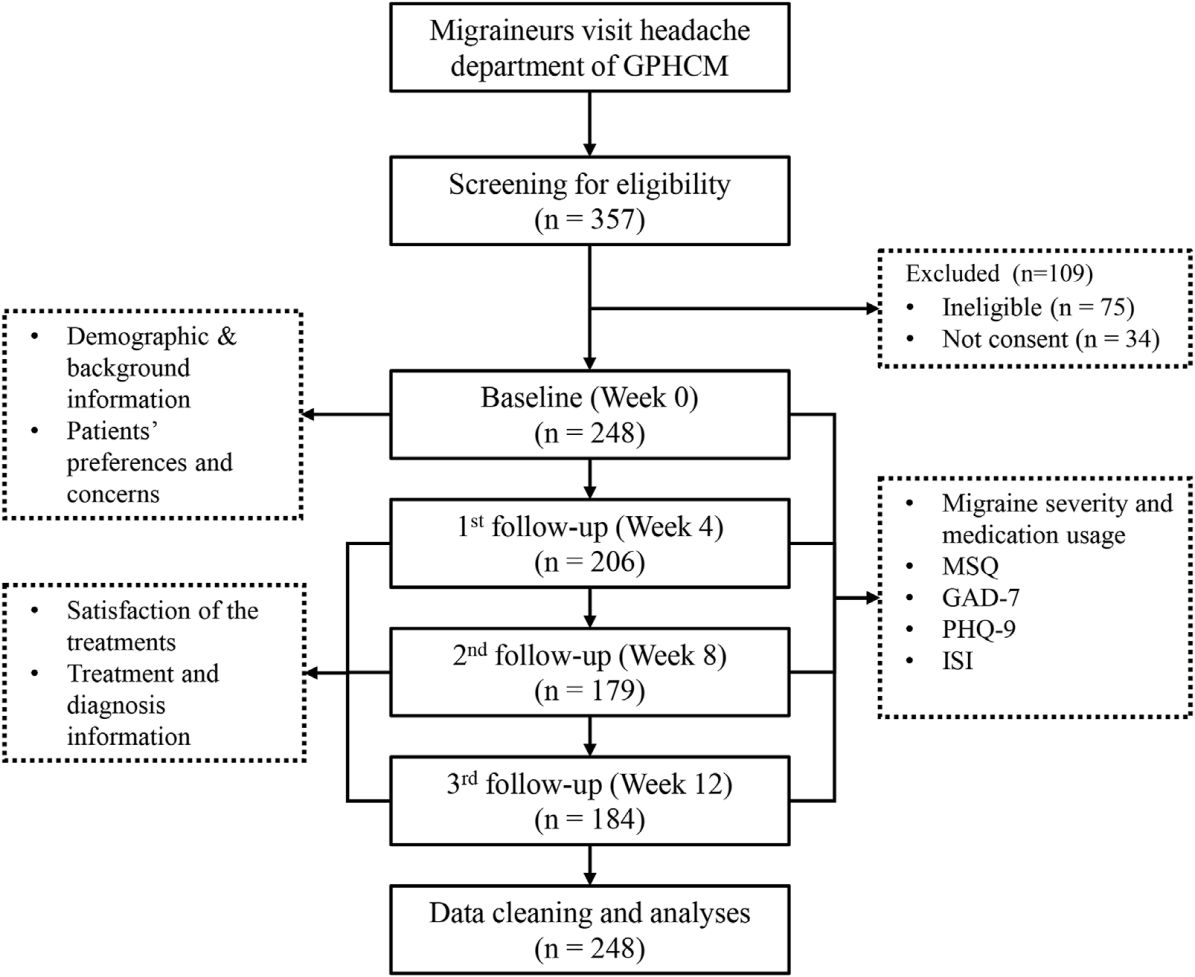
Flowchart of the cohort study.

A total of 248 migraine patients participated in this longitudinal observational study. Among them, 164 participants completed three follow-up assessments at Week 4, Week 8, and Week 12. However, 84 participants missed at least one follow-up evaluation due to personal reasons. Specifically, 206 participants completed assessment at week 4, 179 participants completed assessments at week 8, and 184 individuals completed it at week 12 (Figure 1). It is notably that participation or absence in one follow-up assessment did not determine their involvement status in subsequent assessments. All the 248 participants were included for analyses based on an imputed dataset.

### 3.2 Times of hospital visits and treatment duration

The mean times of hospital visits of the 248 migraine participants was 2.98, and 92 (37.1%) participants visited the hospital only once for their migraines within 12 weeks-observation period. As for treatment duration, 73 participants received CHM treatment for 28 days or more, and they were categorised as subgroup A (CHM ≥28 days). The remaining 175 participants undertook CHM treatment for less than 28 days, therefore were included in subgroup B (CHM <28 days).

### 3.3 Participants’ characteristics at baseline

Notably, participants from subgroup A exhibited a more advanced age and a longer migraine disease duration. In addition, disease severity measured by monthly migraine days, peak pain NRS and monthly migraine frequency in subgroup A significantly surpassed that of subgroup B. Moreover, the frequency of days on acute medications over the 4 weeks preceding the baseline assessment was notably higher in subgroup A in comparison to subgroup B (Table 1).

**TABLE 1:** Baseline characteristics, comorbidities of migraine participants.

			Total (n = 248)	CHM ≥28 days (n = 73)	CHM <28 days (n = 175)	Significance		
Characteristics	Item		Mean (Standard deviation)			df	t	*p*
	Age, years		35.40 (9.34)	37.10 (9.76)	34.70 (9.10)	246	−1.852	0.033*
	Onset age, years		25.44 (9.40)	25.42 (9.34)	25.44 (9.45)	246	0.012	0.495
	Disease duration (years)		9.96 (7.97)	11.67 (7.58)	9.25 (8.04)	211.792	−2.202	0.014*
	Monthly migraine days (days)		6.39 (6.89)	8.05 (8.10)	5.69 (6.20)	108.855	−2.234	0.014*
	Peak pain NRS		7.15 (1.99)	7.51 (2.10)	7.01 (1.93)	246	−1.814	0.035*
	Monthly migraine frequency (valid number = 223)		2.83 (2.24)	3.42 (2.28)	2.58 (2.18)	221	2.560	0.006*
	Migraine duration (hours, valid number = 223)		21.04 (21.68)	20.97 (21.46)	21.06 (21.84)	221	0.029	0.488
	MSQ-RFR		59.07 (20.01)	58.90 (20.04)	59.14 (20.05)	246	0.083	0.467
	MSQ-RFP		67.74 (19.54)	68.42 (17.62)	67.46 (20.33)	246	−0.355	0.362
	MSQ-EF		72.26 (21.78)	73.78 (23.04)	71.62 (21.27)	246	−0.710	0.239
	Generalised anxiety disorder-7 (GAD-7)		5.94 (4.59)	5.67 (4.34)	6.06 (4.69)	246	0.603	0.274
	Patient health quationnaire-9 (PHQ-9)		6.31 (4.83)	6.45 (4.88)	6.26 (4.82)	246	−0.289	0.386
	Insomnia severity index (ISI)		8.14 (5.96)	8.29 (5.69)	8.08 (6.08)	246	−0.250	0.402
	Days taking acute medication over 4 weeks prior to baseline assessment		2.22 (4.20)	3.53 (5.92)	1.67 (3.09)	88.795	−2.553	0.006**
	Item	Categorises	Number (%)			df	χ2	*p*
	Gender	Female	219 (88.31)	65 (89.04)	154 (88.00)	1	0.054	0.816
		Male	29 (11.69)	8 (10.96)	21 (12.00)			
	Aura	Migraine with aura	80 (32.26)	20 (27.40)	60 (34.29)	1	1.119	0.290
		Migraine without aura	168 (67.74)	53 (72.60)	115 (65.71)			
	Family history	Yes	111 (44.76)	31 (42.47)	80 (45.71)	1	0.220	0.639
		No	137 (55.24)	42 (57.53)	95 (54.29)			
	Chronic migraine	Yes	25 (10.08)	9 (12.33)	16 (9.14)	1	0.577	0.448
		No	223 (89.92)	64 (87.67)	159 (90.86)			
	Pure menstrual migraine ^△^ (valid number = 219)	Yes	71 (32.42)	24 (36.90)	47 (30.50)	2	1.940	0.370
		No	122 (55.71)	36 (55.40)	86 (55.80)			
		Unclear	26 (11.87)	5 (7.70)	21 (13.60)			
	Menstrually related migraine ^△^ (valid number = 219)	Yes	120 (54.79)	41 (63.08)	79 (51.30)	2	2.647	0.266
		No	44 (20.09)	10 (15.38)	34 (22.08)			
		Unclear	55 (25.11)	14 (21.54)	41 (26.62)			
	Acute medication use	Yes	151 (60.89)	50 (68.49)	101 (57.71)	1	2.513	0.113
		No	97 (39.11)	23 (31.51)	74 (42.29)			
	Prophylactic medication use	Yes	14 (5.65)	9 (12.30)	5 (2.90)	1	6.989	0.008**
		No	234 (94.35)	64 (87.70)	170 (97.10)			
Comorbidities	Anxiety (by GAD-7 **≥ 5**)^#^	Yes	147 (59.27)	42 (57.53)	105 (60.00)	1	0.130	0.719
		No	101 (40.73)	31 (42.47)	70 (40.00)			
	Depression (by PHQ-9 **≥ 5**)^#^	Yes	152 (61.29)	46 (63.01)	106 (60.57)	1	0.130	0.719
		No	96 (38.71)	27 (36.99)	69 (39.43)			
	Insomnia (by ISI **≥ 7**)^#^	Yes	118 (47.58)	36 (49.30)	82 (46.90)	1	0.125	0.724
		No	130 (52.42)	37 (50.70)	93 (53.10)			

Note: ^#^ The comorbidities were assessed by cutting-off scores of the corresponding scales. * difference is significant at the 0.05 level, ** difference is significant at the 0.01 level, EF: emotional function, GAD-7: Generalised Anxiety Disorder 7-item Scale, ISI: insomnia severity index scale, MSQ: migraine specific quality of life questionnaire, N: number, NRS: numeric rating scale, PHQ-9: Patient Health Questionnaire-9, RFP: role function-preventive, RFR: role function-restrictive.

### 3.4 Preferences and values survey at baseline

Patients’ preferences and values regarding their coming treatments at their initial visits are presented in Table 2. The effect of treatments on migraine severity was unsurprisingly the most popular expectation by 207 (83.47%) of the participants, while the potential side effects of the treatments were concerned by 128 (51.61%) of the participants. In addition, nearly half of the participants expected their treatments to show extended effects in improving sleeping quality (n = 116, 46.77%), regulating psychological status (n = 110, 44.35%) and promoting quality of life (n = 104, 41.94%). Furthermore, 115 (46.37%) of the participants voiced apprehensions regarding the treatment duration. Interestingly, only one third (n = 80, 32.26%) of the participants expected the treatment effects in reducing their usage of acute medications. There was not statistical between-subgroup difference regarding any item of the preferences and values.

**TABLE 2:** Patients’ preferences and values.

Preferences and values	Response	Frequency (%*)			Comparison between subgroups		
		Total (n = 248)	CHM ≥28 days (n = 73)	CHM <28 days (n = 175)	χ^2^	df	*p*
The effects of medications on migraine symptoms	Selected	207 (83.47)	66 (90.41)	141 (80.57)	3.614	1	0.057
	Not selected	41 (16.53)	7 (9.59)	34 (19.43)			
Potential side effects of medications	Selected	128 (51.61)	39 (53.42)	89 (50.86)	0.136	1	0.712
	Not selected	120 (48.39)	34 (46.58)	86 (49.14)			
The effects of medications on sleep quality	Selected	116 (46.77)	35 (47.95)	81 (46.29)	0.057	1	0.811
	Not selected	132 (53.23)	38 (52.05)	94 (53.71)			
Treatment duration	Selected	115 (46.37)	35 (47.95)	80 (45.71)	0.103	1	0.748
	Not selected	133 (53.63)	38 (52.05)	95 (54.29)			
The effects of medications on psychological status	Selected	110 (44.35)	38 (52.05)	72 (41.14)	2.485	1	0.115
	Not selected	138 (55.65)	35 (47.95)	103 (58.86)			
The effects of medications on quality of life	Selected	104 (41.94)	37 (50.68)	67 (38.29)	3.252	1	0.071
	Not selected	144 (58.06)	36 (49.32)	108 (61.71)			
Whether the treatment can reduce acute medication usage	Selected	80 (32.26)	28 (38.36)	52 (29.71)	1.76	1	0.185
	Not selected	168 (67.74)	45 (61.64)	123 (70.29)			
The frequency of taking medication	Selected	60 (24.19)	17 (23.29)	43 (24.57)	0.046	1	0.83
	Not selected	188 (75.81)	56 (76.71)	132 (75.43)			
Out-of-pocket cost caused by the treatment	Selected	60 (24.19)	14 (19.18)	46 (26.29)	1.419	1	0.234
	Not selected	188 (75.81)	59 (80.82)	129 (73.71)			
The convenience of taking medications	Selected	50 (20.16)	10 (13.70)	40 (22.86)	2.684	1	0.101
	Not selected	198 (79.84)	63 (86.30)	135 (77.14)			
Acceptance of the treatment	Selected	34 (13.71)	7 (9.59)	27 (15.43)	1.485	1	0.223
	Not selected	214 (86.29)	66 (90.41)	148 (84.57)			

Note: * percentage within column.

### 3.5 Western medications usage

**FIGURE 2:**
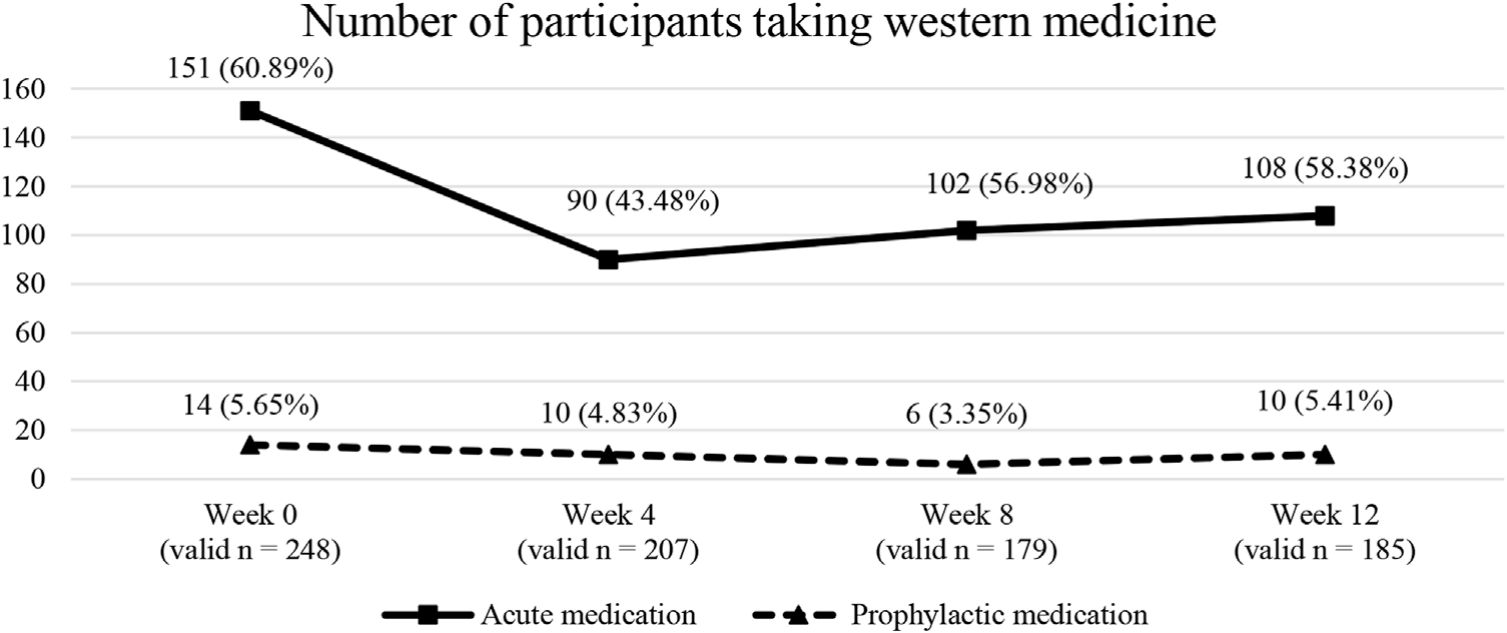
Number of participants taking western medications at different timepoints.

As indicated by Figure 2, the percentage of participants taking acute medications declined from 60.89% at baseline to 43.48% at week 4 but climbed up again to 58.38% at week 12. In contrast, the percentage of participants taking prophylactic medications remained around 5% throughout the observation period.

Detailed descriptions of self-administrated acute medication usage at baseline are presented in Table 3. Among the 151 participants reporting taking acute medications for their migraines at baseline, the most common classification of acute medications was monotherapy of non-migraine specific acute medications, including Ibuprofen (n = 80, 52.98%) and Paracetamol (n = 31, 20.53%). In contrast, migraine specific acute medication, such as triptans (n = 5, 3.31%) and ergotamine (n = 1, 0.66%), were only used by a limited number of migraine participants (Table 3).

**TABLE 3:** Category of acute medications at baseline and the corresponding behaviours.

Category of acute medication				Number (%) (total number = 151)
Single	Non-migraine specific acute medication	NSAIDs	Ibuprofen	80 (52.98%)
			Diclofenac	3 (1.99%)
			Celecoxib	2 (1.32%)
			Aspirin	2 (1.32%)
			Metamizole	2 (1.32%)
			Loxoprofen	2 (1.32%)
		Paracetamol or Panadol		31 (20.53%)
	Migraine-specific acute medication	Triptans		5 (3.31%)
		Ergotamine		1 (0.66%)
Complex (non-migraine specific)	Caffeine complex	Paracetamol, Caffeine and Aspirin Powder		21 (13.91%)
		EVE series		10 (6.62%)
		Compound Aminopyrine Phenacetin Tablets		2 (1.32%)
	Other complex	Others		6 (3.97%)
Behaviours of taking acute medications				
Acute medication adherence		Follow the instructions strictly		118 (78.15%)
		Read the instructions but not followed		13 (8.61%)
		Neither read nor followed the instructions		19 (12.58%)
		Unclear		2 (1.32%)
Receiving professional advice on the acute medications from clinicians or pharmacists		Yes		49 (32.45%)
		No		103 (68.21%)

Note: EVE, series: pain reliever ‘EVE’ was as the first over-the-counter ibuprofen product launched in Japan in 1985. NSAIDs: nonsteroidal anti-inflammatory drugs.

In addition, 78.15% of the 151 participants strictly followed the drug dosage as instructed. However, 13 (8.61%) of them did not adhere to the instructed drug dosage despite having read the instructions, and 19 (12.58%) of them did not read the instructions at all. Moreover, less than one third (32.45%) of these participants received professional advice regarding their usage of acute medication (Table 3).

### 3.6 Effectiveness evaluation

#### 3.6.1 Effects on monthly migraine days and pain intensity

According to the controlled GLMMs results, both changes of monthly migraine days and changes of peak pain NRS at follow-up timepoints from baseline were not significantly different between subgroup A and B (Table 4).

**TABLE 4:** Changes from baseline and the between-subgroup comparisons.

Outcome	Timepoint	Subgroup A (CHM ≥28 days)			Subgroup B (CHM <28 days)			Between-subgroup difference of change	*p*
		Marginal mean	Changes from baseline		Marginal mean	Change from baseline			
			Change (95% CI)	*p*		Change (95% CI)	*p*		
Monthly migraine days	Week 0	4.56	-	-	3.99	-	-	-	-
	Week 4	4.27	−0.28 (−1.17, 0.60)	0.526	3.82	−0.17 (−0.69, 0.34)	0.504	−0.02 (−0.26, 0.22)	0.869
	Week 8	3.75	−0.81 (−1.71, 0.10)	0.082	4.03	0.04 (−0.54, 0.62)	0.886	−0.21 (−0.47, 0.06)	0.124
	Week 12	3.38	−1.18 (−2.08, −0.28)	0.010*	3.64	−0.35 (−0.92, 0.22)	0.230	−0.21 (−0.48, 0.06)	0.130
Peak pain NRS	Week 0	6.64	-	-	6.78	-	-	-	-
	Week 4	6.09	−0.55 (−1.22, 0.12)	0.105	5.61	−1.17 (−1.60, −0.73)	<0.001*	0.10 (−0.02, 0.23)	0.110
	Week 8	5.42	−1.22 (−1.90, −0.53)	0.001*	5.62	−1.16 (−1.63, −0.69)	<0.001*	−0.02 (−0.15, 0.12)	0.825
	Week 12	4.75	−1.89 (−2.57, −1.22)	<0.001*	5.28	−1.50 (−1.97, −1.02)	<0.001*	−0.09 (−0.23, 0.05)	0.227
GAD-7	Week 0	5.80	-	-	6.08	-	-	-	-
	Week 4	5.86	0.06 (−0.96, 1.08)	0.906	5.89	−0.20 (−0.86, 0.47)	0.561	0.04 (−0.16, 0.25)	0.679
	Week 8	4.88	−0.92 (−1.94, 0.10)	0.076	6.15	0.06 (−0.68, 0.80)	0.865	−0.18 (−0.41, 0.04)	0.110
	Week 12	5.50	−0.30 (−1.39, 0.78)	0.582	6.28	0.20 (−0.58, 0.98)	0.618	−0.09 (−0.32, 0.14)	0.463
PHQ-9	Week 0	5.27	-	-	5.67	-	-	-	-
	Week 4	5.35	0.08 (−0.91, 1.07)	0.871	5.50	−0.17 (−0.85, 0.50)	0.616	0.05 (−0.17, 0.27)	0.678
	Week 8	4.56	−0.71 (−1.65, 0.23)	0.141	5.33	−0.34 (−1.03, 0.34)	0.329	−0.08 (−0.31, 0.14)	0.479
	Week 12	4.99	−0.28 (−1.29, 0.73)	0.587	5.29	−0.38 (−1.09, 0.32)	0.285	0.02 (−0.22, 0.25)	0.898
ISI	Week 0	6.47	-	-	6.33	-	-	-	-
	Week 4	7.22	0.75 (−0.49, 2.00)	0.234	6.62	0.30 (−0.44, 1.04)	0.431	0.06 (−0.15, 0.28)	0.548
	Week 8	7.39	0.93 (−0.38, 2.24)	0.165	7.25	0.93 (0.09, 1.76)	0.030*	0 (−0.23, 0.22)	0.982
	Week 12	6.38	−0.08 (−1.34, 1.18)	0.900	6.48	0.16 (−0.66, 0.97)	0.708	−0.04 (−0.27, 0.20)	0.757
MSQ-RFR	Week 0	56.51	-	-	54.98	-	-	-	-
	Week 4	63.42	6.92 (2.46, 11.37)	0.002	65.83	10.84 (7.94, 13.75)	<0.001	−0.06 (−0.15, 0.02)	0.144
	Week 8	66.41	9.90 (4.96, 14.84)	<0.001	68.01	13.02 (9.82, 16.23)	<0.001	−0.05 (−0.15, 0.04)	0.287
	Week 12	69.19	12.68 (7.53, 17.84)	<0.001	71.24	16.26 (12.90, 19.62)	<0.001	−0.06 (−0.15, 0.04)	0.248
MSQ-RFP	Week 0	64.75	-	-	62.40	-	-	-	-
	Week 4	55.56	−9.19 (−15.30, −3.08)	0.003	52.13	−10.28 (−14.06, −6.49)	<0.001	0.03 (−0.09, 0.15)	0.660
	Week 8	69.89	5.14 (−1.62, 11.90)	0.136	73.99	11.58 (7.13, 16.03)	<0.001	−0.09 (−0.21, 0.02)	0.121
	Week 12	72.88	8.13 (1.20, 15.06)	0.022	75.53	13.13 (8.63, 17.63)	<0.001	−0.07 (−0.19, 0.05)	0.230
MSQ-EF	Week 0	70.97	-	-	69.42	-	-	-	-
	Week 4	75.90	4.93 (−0.19, 10.04)	0.059	77.91	8.49 (5.19, 11.80)	<0.001	−0.05 (−0.13, 0.03)	0.246
	Week 8	77.63	6.66 (1.24, 12.08)	0.016	77.69	8.27 (4.81, 11.73)	<0.001	−0.02 (−0.11, 0.06)	0.602
	Week 12	78.64	7.67 (2.14, 13.20)	0.007	80.59	11.18 (7.60, 14.75)	<0.001	−0.05 (−0.13, 0.04)	0.295

Note: Statistical analysis method: generalised linear mixed model; The model was adjusted for the targeted outcome at baseline, age, disease duration, chronic migraine (except for monthly migraine days), time-varying western medications for migraine prevention and time-varying acute medications. * difference is significant at the 0.05 level. CHM: Chinese herbal medicine. 95% CI: 95% confidence interval, EF: emotional function, GAD-7: generalised anxiety disorder-7, scale, ISI: insomnia severity index, MSQ: migraine-specific quality of life questionnaire, NRS: numeric rating scale, PHQ-9: patient health questionnaire-9, RFP: role function-preventive, RFR: role function-restrictive.

Monthly migraine days in the entire cohort declined from week 0 to week 12 (*p* = 0.05), as well as from week 4 to week 12 (*p* = 0.038). These reductions could be primarily attributed to subgroup A, as similar reductions were observed in subgroup A. In contrast, patients within subgroup B did not achieve any reduction in monthly migraine days throughout the 12-week observation period (Table 5).

**TABLE 5:** Pairwise comparisons between timepoints.

Subgroups	Pairwise of timepoints	Monthly migraine days		Peak pain NRS	
		Change between timepoints (95% CI)	*p*	Change between timepoints (95% CI)	*p*
Whole cohort	Week 12 - Week 8	−0.38 (−0.84, 0.07)	0.098	−0.51 (−0.85, −0.17)	0.003
	Week 12 - Week 4	−0.53 (−1.03, −0.03)	0.038	−0.84 (−1.21, −0.46)	<0.001
	Week 12 - Week 0	−0.76 (−1.29, −0.23)	0.005	−1.70 (−2.13, −1.28)	<0.001
	Week 8 - Week 4	−0.15 (−0.63, 0.33)	0.545	−0.32 (−0.69, 0.04)	0.082
	Week 8 - Week 0	−0.38 (−0.91, 0.16)	0.169	−1.19 (−1.61, −0.77)	<0.001
	Week 4 - Week 0	−0.23 (−0.73, 0.27)	0.374	−0.86 (−1.26, −0.46)	<0.001
Subgroup A: CHM ≥28 days	Week 12 - Week 8	−0.37 (−1.09, 0.34)	0.306	−0.68 (−1.22, −0.14)	0.014
	Week 12 - Week 4	−0.89 (−1.73, −0.06)	0.037	−1.34 (−1.96, −0.72)	<0.001
	Week 12 - Week 0	−1.18 (−2.08, −0.28)	0.010	−1.89 (−2.57, −1.22)	<0.001
	Week 8 - Week 4	−0.52 (−1.33, 0.29)	0.205	−0.66 (−1.27, −0.05)	0.033
	Week 8 - Week 0	−0.81 (−1.71, 0.10)	0.082	−1.22 (−1.90, −0.53)	0.001
	Week 4 - Week 0	−0.28 (−1.17, 0.60)	0.526	−0.55 (−1.22, 0.12)	0.105
Subgroup B: CHM <28 days	Week 12 - Week 8	−0.39 (−0.93, 0.15)	0.153	−0.34 (−0.73, 0.06)	0.094
	Week 12 - Week 4	−0.18 (−0.73, 0.38)	0.536	−0.33 (−0.75, 0.09)	0.124
	Week 12 - Week 0	−0.35 (−0.92, 0.22)	0.230	−1.50 (−1.97, −1.02)	<0.001
	Week 8 - Week 4	0.22 (−0.32, 0.76)	0.432	0.01 (−0.39, 0.41)	0.964
	Week 8 - Week 0	0.04 (−0.54, 0.62)	0.886	−1.16 (−1.63, −0.69)	<0.001
	Week 4 - Week 0	−0.17 (−0.69, 0.34)	0.504	−1.17 (−1.60, −0.73)	<0.001

Subgrouping	Pairwise of timepoints	GAD-7		PHQ-9		ISI	
		Change between timepoints (95% CI)	*p*	Change between timepoints (95% CI)	*p*	Change between timepoints (95% CI)	*p*
Whole cohort	Week 12 - Week 8	0.40 (−0.21, 1.01)	0.196	0.20 (−0.37, 0.78)	0.483	−0.89 (−1.62, −0.15)	0.018*
	Week 12 - Week 4	0.00 (−0.68, 0.68)	0.996	−0.29 (−0.90, 0.32)	0.357	−0.48 (−1.25, 0.28)	0.217
	Week 12 - Week 0	−0.06 (−0.75, 0.62)	0.854	−0.33 (−0.95, 0.29)	0.300	0.04 (−0.71, 0.79)	0.921
	Week 8 - Week 4	−0.40 (−1.00, 0.20)	0.191	−0.49 (−1.07, 0.09)	0.095	0.41 (−0.35, 1.16)	0.290
	Week 8 - Week 0	−0.47 (−1.11, 0.18)	0.159	−0.53 (−1.13, 0.06)	0.079	0.93 (0.14, 1.71)	0.020*
	Week 4 - Week 0	−0.07 (−0.68, 0.55)	0.835	−0.04 (−0.65, 0.57)	0.891	0.52 (−0.20, 1.24)	0.158
Subgroup A: CHM ≥28 days	Week 12 - Week 8	0.62 (−0.31, 1.55)	0.192	0.43 (−0.49, 1.34)	0.360	−1.01 (−2.23, 0.22)	0.107
	Week 12 - Week 4	−0.37 (−1.46, 0.73)	0.510	−0.36 (−1.36, 0.64)	0.477	−0.83 (−2.15, 0.48)	0.212
	Week 12 - Week 0	−0.30 (−1.39, 0.78)	0.582	−0.28 (−1.29, 0.73)	0.587	−0.08 (−1.34, 1.18)	0.900
	Week 8 - Week 4	−0.99 (−1.95, −0.02)	0.045*	−0.79 (−1.71, 0.14)	0.095	0.17 (−1.13, 1.47)	0.795
	Week 8 - Week 0	−0.92 (−1.94, 0.10)	0.076	−0.71 (−1.65, 0.23)	0.141	0.93 (−0.38, 2.24)	0.165
	Week 4 - Week 0	0.06 (−0.96, 1.08)	0.906	0.08 (−0.91, 1.07)	0.871	0.75 (−0.49, 2.00)	0.234
Subgroup B: CHM <28 days	Week 12 - Week 8	0.14 (−0.59, 0.86)	0.714	−0.04 (−0.70, 0.61)	0.899	−0.77 (−1.57, 0.03)	0.060
	Week 12 - Week 4	0.40 (−0.38, 1.17)	0.315	−0.21 (−0.89, 0.47)	0.547	−0.14 (−0.95, 0.67)	0.732
	Week 12 - Week 0	0.20 (−0.58, 0.98)	0.618	−0.38 (−1.09, 0.32)	0.285	0.16 (−0.66, 0.97)	0.708
	Week 8 - Week 4	0.26 (−0.41, 0.93)	0.449	−0.17 (−0.82, 0.49)	0.618	0.63 (−0.17, 1.43)	0.122
	Week 8 - Week 0	0.06 (−0.68, 0.80)	0.865	−0.34 (−1.03, 0.34)	0.329	0.93 (0.09, 1.76)	0.030*
	Week 4 - Week 0	−0.20 (−0.86, 0.47)	0.561	−0.17 (−0.85, 0.50)	0.616	0.30 (−0.44, 1.04)	0.431

Subgrouping	Pairwise of timepoints	MSQ-RFR		MSQ-RFP		MSQ-EF	
		Change between timepoints (95% CI)	*p*	Change between timepoints (95% CI)	*p*	Change between timepoints (95% CI)	*p*
Whole cohort	Week 12 - Week 8	3.01 (0.04, 5.97)	0.047*	2.29 (−2.07, 6.64)	0.304	1.95 (−1.24, 5.15)	0.231
	Week 12 - Week 4	5.60 (2.42, 8.77)	0.001*	20.38 (16.43, 24.33)	<0.001*	2.71 (−0.64, 6.07)	0.113
	Week 12 - Week 0	14.47 (11.35, 17.59)	<0.001*	10.63 (6.46, 14.80)	<0.001*	9.42 (6.10, 12.74)	<0.001*
	Week 8 - Week 4	2.59 (−0.25, 5.43)	0.074	18.09 (14.22, 21.97)	<0.001*	0.76 (−2.38, 3.90)	0.635
	Week 8 - Week 0	11.46 (8.48, 14.44)	<0.001*	8.34 (4.25, 12.43)	<0.001*	7.47 (4.24, 10.70)	<0.001*
	Week 4 - Week 0	8.87 (6.16, 11.59)	<0.001*	−9.75 (−13.34, −6.16)	<0.001*	6.71 (3.62, 9.79)	<0.001*
Subgroup A: CHM ≥28 days	Week 12 - Week 8	2.79 (−2.12, 7.69)	0.265	2.99 (−4.16, 10.14)	0.412	1.01 (−4.32, 6.34)	0.709
	Week 12 - Week 4	5.77 (0.54, 11.00)	0.031*	17.32 (10.82, 23.82)	<0.001*	2.74 (−2.82, 8.31)	0.333
	Week 12 - Week 0	12.68 (7.53, 17.84)	<0.001*	8.13 (1.20, 15.06)	0.022*	7.67 (2.14, 13.20)	0.007*
	Week 8 - Week 4	2.98 (−1.72, 7.68)	0.213	14.33 (7.97, 20.69)	<0.001*	1.73 (−3.51, 6.98)	0.517
	Week 8 - Week 0	9.90 (4.96, 14.84)	<0.001*	5.14 (−1.62, 11.90)	0.136	6.66 (1.24, 12.08)	0.016*
	Week 4 - Week 0	6.92 (2.46, 11.37)	0.002*	−9.19 (−15.30, −3.08)	0.003*	4.93 (−0.19, 10.04)	0.059
Subgroup B: CHM <28 days	Week 12 - Week 8	3.23 (−0.03, 6.50)	0.052	1.55 (−3.30, 6.40)	0.531	2.90 (−0.60, 6.41)	0.104
	Week 12 - Week 4	5.41 (1.92, 8.91)	0.002*	23.41 (19.11, 27.70)	<0.001*	2.68 (−1.01, 6.37)	0.154
	Week 12 - Week 0	16.26 (12.90, 19.62)	<0.001*	13.13 (8.63, 17.63)	<0.001*	11.18 (7.60, 14.75)	<0.001*
	Week 8 - Week 4	2.18 (−0.95, 5.31)	0.172	21.86 (17.61, 26.11)	<0.001*	−0.22 (−3.66, 3.21)	0.899
	Week 8 - Week 0	13.02 (9.82, 16.23)	<0.001*	11.58 (7.13, 16.03)	<0.001*	8.27 (4.81, 11.73)	<0.001*
	Week 4 - Week 0	10.84 (7.94, 13.75)	<0.001*	−10.28 (−14.06, −6.49)	<0.001*	8.49 (5.19, 11.80)	<0.001*

Note: Statistical analysis method: generalised linear mixed model; The model was adjusted for the targeted outcome at baseline, age, disease duration, chronic migraine (except for monthly migraine days), time-varying western medications for migraine prevention and time-varying acute medications. * difference is significant at the 0.05 level. CHM: Chinese herbal medicine. 95% CI: 95% confidence interval, EF: emotional function, GAD-7: generalised anxiety disorder-7, scale, ISI: insomnia severity index, MSQ: migraine-specific quality of life questionnaire, NRS: numeric rating scale, PHQ-9: patient health questionnaire-9, RFP: role function-preventive, RFR: role function-restrictive.

In terms of the peak pain NRS, there was a consistent and prolonged downward trend observed within the entire cohort and either subgroup. Notably, the reduction in peak pain NRS scores within subgroup A was sustained not only from week 0 to the subsequent follow-up timepoints, but also from week 4 to week 8 and week 12, as well as persisting from week 8 to week 12. In contrast, the deduction of peak pain NRS scores in subgroup B was not consistently maintained from week 4 to week 8 or week 12, nor from week 8 to week 12 (Table 5).

#### 3.6.2 Effects on migraine comorbidities

As Table 4 indicated, changes of GAD-7, PHQ-9 and ISI scores at follow-up timepoints from week 0 were not significantly different between subgroups.

In terms of PHQ-9, no significant variations were observed across the entire cohort or within either of the subgroups.

Regarding GAD-7, the scores remained consistent throughout the observation period for the entire cohort and subgroup B. However, within subgroup A, a significant reduction in GAD-7 scores emerged from week 4 to week 8 (*p* = 0.045).

As for ISI, the score within the entire cohort exhibited a significant increase from week 0 to week 8 (*p* = 0.020), followed by a reduction from week 8 to week 12 (*p* = 0.018). The substantial deterioration in ISI scores from week 0 to week 8 was primarily attributed to subgroup B (*p* = 0.03), whereas the ISI score in subgroup A did not significantly increased (Table 5).

#### 3.6.3 Effects on migraine-specific quality of life

Both subgroups gained a steady increase in MSQ-RFR from week 0 to each follow-up timepoint, and they also achieved a significant increase in MSQ-RFP and MSQ-EF from week 0 to week 12. However, only subgroup B exhibited an increase in MSQ-RFP from week 0 to week 8, and an increase in MSQ-EF from week 0 to week 4, whereas these changes were not observed in subgroup A (Table 5). Nevertheless, the changes of MSQ-RFR, MSQ-RFP and MSQ-EF scores at follow-up timepoints from baseline were not significantly different between subgroups (Table 4).

### 3.7 Adverse events

Patient-reported adverse events (AEs) were collected from CRFs. Throughout the observation period, 30 participants reported a total of 51 AEs, involving 17 symptoms. The most common AE reported was discomfort in the stomach (n = 18), followed by diarrheal (n = 8), fatigue (n = 4) and dizziness (n = 4). No severe AEs were reported. Furthermore, the number of participants reporting AEs and the number of AE cases appeared comparable between the two subgroups based on CHM treatment duration (≥28 days vs. < 28 days) (Table 6). No statistical difference was detected between subgroup A (CHM treatment duration ≥28 days) and subgroup B (CHM treatment duration <28 days) regarding the number of patients reporting AEs (χ^2^ = 3.174, *p* = 0.075).

**TABLE 6:** Adverse events reported by participants.

Adverse event	Week 0	Week 4	Week 8	Week 12	Total number of cases
Discomfort in the stomach	13 (7 vs. 6)	1 (0 vs. 1)	2 (2 vs. 0)	2 (1 vs. 1)	18 (10 vs. 8)
Diarrheal	0	6 (4 vs. 2)	2 (1 vs. 1)	0	8 (5 vs. 3)
Fatigue	1 (1 vs. 0)	1 (0 vs. 1)	1 (0 vs. 1)	1 (0 vs. 1)	4 (1 vs. 3)
Elevated blood pressure	0	1 (1 vs. 0)	0	0	1 (1 vs. 0)
Headache	0	1 (1 vs. 0)	0	0	1 (1 vs. 0)
Dizziness	2 (0 vs. 2)	2 (1 vs. 1)	0	0	4 (1 vs. 3)
Poor appetite	1 (1 vs. 0)	1 (0 vs. 1)	0	0	2 (1 vs. 1)
Skin rash	0	1 (0 vs. 1)	0	0	1 (0 vs. 1)
Abnormal liver function	0	1 (0 vs. 1)	0	0	1 (0 vs. 1)
Oral ulcer	0	1 (0 vs. 1)	0	0	1 (0 vs. 1)
Pollakisurie	2 (1 vs. 1)	1 (1 vs. 0)	0	0	3 (2 vs. 1)
Abdominal pain	0	1 (1 vs. 0)	0	0	1 (1 vs. 0)
Gingival bleeding	0	1 (1 vs. 0)	0	0	1 (1 vs. 0)
Hypomenorrhea	0	1 (1 vs. 0)	0	0	1 (1 vs. 0)
Abdominal distention	0	1 (0 vs. 1)	0	0	1 (0 vs. 1)
Palpitation	2 (1 vs. 1)	0	0	0	2 (1 vs. 1)
Insomnia	1 (1 vs. 0)	0	0	0	1 (1 vs. 0)
Total cases of AEs	22 (12 vs. 10)	21 (11 vs. 10)	5 (3 vs. 2)	3 (1 vs. 2)	51 (25 vs. 26)
Total number of participants reporting AEs*	20 (11 vs. 9)	16 (7 vs. 9)	5 (3 vs. 2)	3 (0 vs. 3)	30 (13 vs. 17)

Note: AE: adverse event; vs.: number of AE, cases in subgroup A vs. number of AE, cases in subgroup B; * number of participants reporting AEs.

### 3.8 Herbs and patented Chinese herbal medicine products for migraine

The medical records of the responders (≥50% reduction in monthly migraine days at week 12) were retrieved for treatment pattern analysis.

#### 3.8.1 Frequency analysis of herbs and their mechanisms

A total of 341 herbal prescriptions involving 147 herbs were eligible for analyses. The most commonly used herb was *gan cao*, which was prescribed 315 times, followed by *gui zhi* (n = 265) and *chuan xiong* (n = 246). The remaining herbs with top ten frequency included *fu ling* (n = 241), *bai zhu* (n = 231), *yan hu suo* (n = 222), *ban xia* (n = 220), *chen pi* (n = 218), *bai shao* (n = 210), and *xiang fu* (n = 204).

#### 3.8.2 Frequency analysis of patented Chinese herbal medicine products

Patented Chinese herbal medicine products (PCHMPs) used for migraines were presented in Table 7. Interestingly, the most commonly used PCHMPs were *Wei su* granule (n = 75) and *Jian wei yu yang* tablet (n = 73), which mainly targeted at gastrointestinal conditions. Headache-specific PCHMPs like *Tian shu* tablet and *Tong tian* oral solution were prescribed by less than 10% of the cases.

**TABLE 7:** Frequency and functions of commonly used PCHMPs.

Names	Frequency	Functions	Targeted diseases
*Wei su* granule	75	Regulating *qi*, reducing distention in Stomach and alleviating stomach-ache	Chronic gastritis and gastroduodenal ulcer
*Jian wei yu yang* tablet	73	Tonifying the Spleen, smoothing the Liver and alleviating pain	Stomach-ache, gastroduodenal ulcer
*San qi tong shu* capsule	28	Activating circulation of Blood and smoothing the meridians	Cardio-cerebral vascular atherosclerosis
*Jing tong* granule	28	Activating circulation of qi and Blood, removing the Blood stasis and alleviating pain	Cervical spondylotic radiculopathy
*Zao ren an shen* capsule	26	Nourishing Blood and calming the spirit	Insomnia, memory loss, dizziness
*Tian shu* tablet	23	Activating circulation of Blood, calming the Liver, smoothing the meridians and alleviating the pain	Headache
*Tong tian* oral solution	18	Removing Blood stasis, activating circulation of Blood, expelling the Wind and alleviating the pain	Migraine
*Xiao yao* pill	16	Tonifying the Speen, nourishing the Blood and smoothing the Liver	Emotional condition with poor appetite and irregular menstruation
*Er shi wu wei shan hu* capsule	15	Clearing the orifices, smoothing the meridian, and alleviating pain	Neurologic conditions including numbness, dizziness, headache, epilepsy, etc.
*Yang xue qing nao* granule	10	Nourishing Blood and calming Liver *yang,* activating the circulation of Blood	Headache, dizziness, insomnia with Chinese medicine syndrome of Liver *yang* uprising and Blood deficiency

Note: *Percentage among patients who were prescribed with PCHMP/WM, for their migraine (n = 271). Functions and targeted conditions of the PCHMPs, reference to Chinese Pharmacopoeia 2020.

## 4 Discussion

### 4.1 Summary and interpretation of the key results

#### 4.1.1 Patients’ characteristics

The mean age of the migraine participants in this study is 35.40 (±9.34) years, falling within the age range with the highest prevalence of migraines (Safiri et al., 2022). Chronic migraine constitutes over 10% of the included participants, a percentage similar to that of chronic migraine among the migraine population in United State (13.65%) (Buse et al., 2021).

The female-to-male ratio in this observational study (7.5: 1) surpasses the general migraine population ratio (3–4:1) (Vetvik and MacGregor, 2017; Lipton et al., 2018), rendering the findings of this study particularly relevant to female migraine patients. This higher percentage of female participants may be attributed to the fact that female participants tend to experience more severe and disabling migraine attacks (Neumeier et al., 2021). In addition, the female-predominance aligns with findings from a previous report, which concluded that female migraine patients were more likely to be Chinese medicine users (Chang et al., 2014).

Aura was reported in over 32% of the migraine participants in this study, which is higher than the occurrence among the general migraine populations (25%) (Rasmussen and Olesen, 1992). Migraine with aura was reported to be associated with reluctant response to conventional pharmacotherapies (Hansen et al., 2015; Hansen and Charles, 2019), which might explain the higher proportion of migraine with aura in the Chinese medicine hospital.

Anxiety, depression and insomnia are the most common comorbidities among the migraine participants, consistent with previous reports (Caponnetto et al., 2021; Lyu et al., 2022b). Notably, depression was comorbid in 61.29% of the migraine participants in this study, the percentage is higher than that among the general migraine population (16%–18%) (Yong et al., 2012; Lee et al., 2020). An increased likelihood of comorbid depression was reported among migraine patients visiting a headache clinic (Amoozegar et al., 2017). Additionally, it was noted that this comorbid depression was inadequately treated (Amoozegar et al., 2017).

#### 4.1.2 Patients’ preferences and values

Patients’ preferences and values encompass the distinct understandings, individual preferences, concerns, expectations and life circumstances (Straus et al., 2018). Values refer to a patient’s attitudes and perceptions regarding various healthcare alternatives, while preferences represent their favoured choices after accounting for their values (Llewellyn-Thomas and Crump, 2013). Established methods to investigate patients’ preferences and values include interviews, focus groups, observation, surveys, narrative description, etc. (Michael et al., 2022). This study employed a combination of behavioural observations, treatment utilisation patterns analysis, and targeted surveys focusing on narrative preferences and values. These approaches collectively facilitated a comprehensive exploration of the diverse spectrum of migraine patients’ preferences and values.

##### 4.1.2.1 Utilisation patterns of acute medications

More than 60% of the migraine participants in the study reported using acute medications for their migraines prior to their initial treatments in the studied Chinese hospital. However, over 60% of them utilised non-specific migraine acute medications like nonsteroidal anti-inflammatory drugs (NSAIDs), while migraine-specific acute medications like triptans was used by less than 5% of the patients. The discrete percentages of patients taking migraine-specific and non-specific acute medications closely resembled findings from a prior report (Zhao et al., 2023). The potential reasons contributing to the high prevalence of NSAIDs mainly involve their low prices and high patient accessibility as over-the-counter medications (Zhao et al., 2023). In contrast, the low patient accessibility and limited choices of drugs restricted the limited spread of triptans among migraine patients (Zhao et al., 2023). Low application of triptans and low adherence to the therapeutic guideline was also reported in Denmark and the United States (Marmura et al., 2015; Lipton et al., 2022; Olesen et al., 2022).

Additionally, up to 68% of the participants did not receive professional advice on their acute medications for migraine, and over one third of them reported poor adherence to the medication instructions. Inadequate consultation rate was reported in the United States, with 27.6% of episodic migraine and 40.8% of chronic migraine responders (Buse et al., 2021). It is appealed to increase consultation and diagnosis rates, as well as promoted patient education, to improve the delivery of appropriate guideline-based treatment, and avoidance of medication overuse (Buse et al., 2021; Katsuki et al., 2023).

##### 4.1.2.2 Factors for the choice of Chinese herbal medicine

Earlier research has indicated that patients possessing multiple comorbidities, complex symptoms, residing in rural areas, being females, and being in the middle to old age group, were more likely to receive CHM treatment (Chang et al., 2014; Lin et al., 2015; Xin et al., 2020). The present study has shed light on the fact that participants undergoing a longer duration CHM therapy in the studied hospital were experiencing more severe migraines, and they tended to be older in age with a longer disease duration of migraine.

##### 4.1.2.3 Treatment duration and times of hospital visits

Within the 12-week observation period, the mean CHM treatment duration was found to be 20.72 days, and the mean frequency of hospital visits was only 2.98 times. In contrast, the mean primary care physical visits for migraine within 3 months was reported to be 2.57 for chronic migraine and 2.54 for episodic migraine in Europe, and the corresponding neurologist specialist visits was 1.53 and 1.73, respectively (Bloudek et al., 2012). In this study, the limited treatment duration and times of hospital visits might have been interrupted by the quarantine of COVID-19 (Buse et al., 2022; Jokubaitis et al., 2023). Low adherence and persistence for prophylactic migraine medication were reported to be associated with low response rate and unwanted side effects (Lafata et al., 2010; Hepp et al., 2014; Rimmele et al., 2023). CHM has been identified as an effective therapy for migraine with an adequate treatment duration, and the side effects were mild. The positive treatment effects of CHM at a sufficient treatment duration may be advertised to the migraine patients to increase the adherence and persistence.

##### 4.1.2.4 Patients’ narrative preferences and values

The survey for patients’ preferences and values in this study was refined based on commonly reported items in previous studies and new insights from our previous research (Wenzel et al., 2004; Kelman, 2006; Rozen, 2006; Peres et al., 2007; Mansfield et al., 2019; Lyu et al., 2022b). Consistent with previous reports, increased efficacy was the most reported expectation, while side effects were the top concern, among the migraine participants (Kelman, 2006; Rozen, 2006; Peres et al., 2007; Mansfield et al., 2019). Improved quality of life after treatments was also expected by migraine patients (Kelman, 2006). No statistical difference was observed between subgroups regarding any item of preferences and values. It is novel to reveal that a substantial percentage of migraine participants expected the extended effects on migraine comorbidities of anxiety, depression, and insomnia in our report, while migraine comorbidities issues were seldom addressed by previous studies. In addition, as the increased concern about medication overuse, our study incorporated a novel option regarding reducing acute medication usage. The result indicated that limited number of participants expressed expectation to reduce their acute medications, as the number of days taking acute medication at baseline was merely 2.22/4 weeks. Near half of the migraine participants showed attention to treatment duration, meanwhile around 75% of the participants discontinued their treatments within 28 days. The relationship between treatment effect and treatment duration needs further investigation.

#### 4.1.3 Effectiveness of Chinese herbal medicine for migraine

As indicated by the outcomes derived from the GLMM analyses above, CHM appeared beneficial in reducing monthly migraine days and peak pain NRS. Particularly, the beneficial effects required a minimum of 28 days CHM treatment. Furthermore, when administered for 28 days or more, CHM might lead to a more sustained effects in reducing peak pain NRS, compared to that when administered for less than 28 days. In summary, a longer CHM treatment duration is associated with a better treatment response, this is consistent with the conclusions from our previous systematic review (Lyu et al., 2022a).

In addition, a minimum duration of CHM treatment for 28 days appears to be beneficial for reducing migraine-comorbid anxiety, and preventing the worsening of insomnia, but it has limited effect on depression. However, it is important to note that depression and insomnia might have been affected by the stress caused by the unanticipated COVID-19 pandemic (Suzuki et al., 2021; Buse et al., 2022; Thaxter and Smitherman, 2022), which was not systematically assessed in this study. Clinical evidence has found that several CHM formulae were effective in controlling anxiety, depression and insomnia, either as independent conditions or as comorbidities of other conditions such as heart failure, chronic obstructive pulmonary disease, etc. (Hu et al., 2021; Yang et al., 2021; Wang et al., 2022). The extended effects of CHM on the psychological comorbidities of migraine need further examination.

Moreover, CHM may effectively improve specific quality of life for the migraine patients, regardless of the treatment duration. The beneficial effects of CHM in improving patients’ quality of life were also reported in our previous systematic reviews for migraines (Lyu et al., 2020; Lyu et al., 2022a), and for other conditions, such as atopic dermatitis, cancer, perimenopausal women, etc. (Hon et al., 2007; Chan et al., 2011; Xia et al., 2012).

These effectiveness evaluations in return addressed the leading expectations of the participants in this study, which were also in consistent with previous reports on patients’ preferences and values (Kelman, 2006; Rozen, 2006; Peres et al., 2007; Mansfield et al., 2019).

Acute medication overuse is a major but modifiable risk factor for chronic migraine (Buse et al., 2019). Approximately 60% of the migraine participants took acute medications for their migraines at baseline. Notably, the proportion of participants taking acute medication reduced significantly at week 4 but increased at around 58% towards the end of the observation period. Previously, CHM-induced reduction in acute medications for chronic tension-type headaches was reported in another observational study (Tong et al., 2015). The reduction in acute medication usage and the potential of CHM for preventing and reversing medication overuse headache warranted more robust investigation.

#### 4.1.4 Safety profile

Throughout the 12-week observation period, a total of 51 AEs were documented by 30 (12.10%) of the participants. The incidence rate of AEs was comparatively lower than those reported for erenumab (37%) and onabotulinumtoxinA (25%) based on real-world observations (Matharu et al., 2017; Schenk et al., 2022). In addition, among the instances, 18 (35.29%) of the patient-reported AEs were gastrointestinal discomforts, and these discomforts were also the most commonly reported AEs in previous reports (Schenk et al., 2022; Silberstein et al., 2023). Moreover, no severe AEs were reported in the current study. The safety profile of CHM for migraine, including the low rate of AEs and the prevailing occurrence of gastrointestinal discomforts, was consistent with our previous findings (Lyu et al., 2020; Lyu et al., 2022a).

#### 4.1.5 Chinese herbal medicine utilisation patterns for migraine

The frequency analysis indicated that herbs *gan cao*, *gui zhi*, *chuan xiong*, *fu ling*, *bai zhu*, *yan hu suo*, *ban xia*, *chen pi*, *bai shao* and *xiang fu*, were widely utilised for migraine, which is similar to the findings of our previous research (Lyu et al., 2020; Lyu et al., 2022a; Lyu et al., 2022b; Zhang et al., 2022). These herbs were reported to exhibit anti-migraine, anti-depression, neuroprotective, sedative-hypnotic and/or antiemetic actions, as summarised in Table 8. These effects not only address the migraine headaches, but also benefit migraine-associated comorbidities including depression and insomnia, as well as accompany symptoms such as nausea and vomiting. Specifically, the anti-migraine actions are frequently linked to the modulation of monoamine neurotransmitters (e.g., 5-HT, CGRP) and their turnover rates, as investigated in the case of *chuan xiong* (Wang et al., 2011; Pu et al., 2019).

**TABLE 8:** Potential mechanisms of frequent herbs for migraine and associated comorbidities.

Name in *pin yin*	Scientific name*	Preparation	Chemical constituents	Subject	Administration	Bioactivity	Mechanism of action
*Gan cao*	1. *Glycyrrhiza uralensis* Fisch.	Compound	Glycyrrhizin	Lithium-pilocarpine-induced status epilepticus rat	Intravenous injection	Neuroprotective effects	Inhibiting HMGB1 and protecting blood brain barrier permeability Li et al. (2019).
	2. *Glycyrrhiza inflata* Batalin	Compound	Glycyrrhizin	CUMS mice	Intragastric administered	Antidepressant effects	Regulating enzyme of the kynurenine pathway Wang et al. (2018).
	3. *Glycyrrhiza glabra* L.	Extract (ethanol)	Glycyrrhizin	Ischemic stroke mice	Intragastric administered	Neuroprotective effects	Regulating inflammation-related neuronal cells like microglia and astrocytes Choi et al. (2022).
*Rou gui*	*Cinnamomum cassia* (L.) J. Presl	Cinnamon powder	N/A	Migraine patients	Oral administration	Anti-migraine effects	Deducing the serum concentrations of IL-6 and NO of migraine patients Zareie et al. (2020).
*Chuan xiong*	*Ligusticum striatum* DC.	Compound	Senkyunolide I	Nitroglycerin-induced migraine rat	Intragastric administered	Anti-migraine effects	Adjusting the levels of monoamine neurotransmitters and their turnover rates, decreasing NO levels in the blood and brain Wang et al. (2011).
		Compound	Ligustrazine	Nitroglycerin-induced migraine rat	Intravenous injection	Anti-migraine effects	Inhibiting over-expression of P2X3, TRPV1, c-fos, and ERK Li et al. (2021a).
		Compound	Alkaloids	Nitroglycerin-induced migraine rat Reserpine-induced migraine mice	Orally administrated	Anti-migraine effects	Increasing the levels of 5-HT and 5-HIAA in the brain, regulating the expression of monoamine neurotransmitter 5-HT_1B_ receptor and *c*-*Jun* in the periaqueductal gray Pu et al. (2019).
		Extract (supercritical carbon dioxide)	Volatile oil from Rhizoma Ligustici *Chuanxiong* Hort.	Nitroglycerin-induced headache mice and rat	Oral administration, Administered intraperitoneally	Anti-headache effects	Increasing the level of plasma ET, inhibiting the c-fos gene expression in the brain stem and hypothalamus and the level of plasma CGRP Peng et al. (2009).
*Fu ling*	*Poria cocos* (Schw.) Wolf	Compound	Acidic polysaccharides	CUMS rat	Intragastric administration	Antidepressant effects	Regulating neurotransmitters and NLRP3 inflammasome signalling pathway Chen et al. (2021).
		Compound	Pachymic Acid	Pentobarbital-induced sleep mice	Intragastric administration	Sedative-hypnotic effects	Enhancing pentobarbital-induced sleeping behaviors via GABAA-ergic mechanisms in rodents Shah et al. (2014).
		Compound	Pachymic acid	Cerebral ischemia/reperfusion injury rat	Intragastric administration	Neuroprotective effects	Activating PI3K/Akt signalling pathway Pang et al. (2020).
		Extract (ethanol)	N/A	ACTH-induced sleep disturbed mice	Intragastric administration	Sedative-hypnotic effects	Improving sleep quality under a normal sleep state through the GABA_A_ receptor; promoting and improving sleep quality and sleep structure in both the arousal activation state and stress- based sleep disturbance Kim et al. (2022).
		Extract (water)	Polysaccharides	CUMS rat	Intragastric administration	Antidepressant-like effects	Regulating monoaminergic neurotransmission (DA, 5-HT) and inactivation of inflammation (p38, NF-κb and TNF-α) Huang et al. (2020).
*Bai zhu*	*Atractylodes macrocephala* Koidz.	Compound	Atractylenolide III	CUMS rat	Intragastric administration	Antidepressant- and anxiolytic-like effects	Inhibiting hippocampal neuronal inflammation (proinflammatory cytokines levels) Zhou et al. (2021).
		Compound	Atractylenolide I	CUMS mice	Intragastric administration	Antidepressant effects	Inhibiting NLRP3 inflammasome activation to decrease IL-1β production Gao et al. (2018).
		Compound	Atractylenolide III	1. Transient occlusion to the middle cerebral artery mice	Intragastric administration, *In vitro*	Neuroprotective and anti-neuroinflammatory effects	Inhibiting neuroinflammation, partly by JAK2/STAT3-dependent mitochondrial fission in microglia Zhou et al. (2019).
				2. Oxygen glucose deprivation-reoxygenation stimulated primary microglia from mice			
*Yan hu suo*	*Corydalis yanhusuo* W. T. Wang ex	Compound	Tetrahydropalmatine, corydaline, protopine, dehydrocorydaline	Formalin-induced pain mice; Nav1.7-CHO cells and Nav1.5-CHO cells	Intragastric administration, *In vitro*	Analgesic effects	Increasing the level of creatine kinase-MB and inhibiting the peak currents, promoting the activation and inactivation phases of Nav1.5 and Nav1.7 Xu et al. (2021).
		Compound	Total alkaloids (Glaucine, Dehydrocorydaline, Palmatine, l-THP, Berberine, Corydaline, Tetrahydrocoptisine, Protopine, Tetrahydroberberine)	CCI-induced neuropathic pain rat	Intragastric administration	Anti-neuropathic pain effects	Relieving neuropathic pain in chronic constriction injury rats and repressing spinal central sensitization Zhou et al. (2023).
		Extract (ethanol)	Corydalis tuber	CCI-induced neuropathic pain rat	Intragastric administration	Antinociceptive effects	Decreasing the nerve injury-induced mechanical allodynia, alleviating thermal heat hyperalgesia, reducing the nerve injury-induced phosphorylation of NMDA receptor NR1 subunit in the spinal dorsal horn Choi et al. (2012).
		Extract (water)	Dehydrocorybulbine, L-tetrahydropalmatine	Male dopamine D2 receptor knockout mice	Orally administrated	Antinociceptive effects	Mediating dopamine D2 receptor ant agonism Wang et al. (2016).
*Ban xia*	*Pinellia ternate* (Thunb.) Makino	Granules tuber of Pinellia Ternate (Thunb.) Breit.	L-arginine, Aspartic acid, 9-Oxo-nonanoic acid, r-Aminobutyric acid, Alanine, Proline, Coniferin, 5-Hydroxymethylfurfural, 5-Methyl uracil, Caffeic acid, 1,6:3,4-Dianhydro-β-D-allosep, Sucrose, Glutamic acid, Valine, 6- purine, Tyrosine, Methionine, Isoleucine, Pedatisectine B, Phenylalanine, Threonine, 5-Amyl-2-pyrone, Adenosine, Cyclo-(Val-L-Tyr), 1-O-glucosyl-N-2′-palmitoyl-4,8-sphingodienine, 2-Ethenyl butenal, Vanillic acid, Chrysophanol, Methyl phenanthrene, Inosine, Pinellic acid, Gingerol	Male SPF C57BL/6J mice	Intragastric administration	Sedative and hypnotic effects	Increasing rapid eye movement (REM) sleep and non-REM (NREM) sleep while decreasing wakefulness, decreasing the number of bouts of wakefulnes and increasing the number of bouts of NREM sleep Lin et al. (2019).
		Raw Pinelliae Rhizoma		Male C57BL/6 J mice	Intragastric administration	Sedative and hypnotic effects	Raw Pinelliae Rhizoma increased REM sleep duration in both the light and dark phases and increased the number of transitions both from NREM sleep to REM sleep and from REM sleep to wakefulness Lin et al. (2023).
		Extract of *ban xia* and *sheng jiang* at a ratio of 2:1	Ephedrine, succinic acid, 6-gingerol, and 6-shogaol	Rat Pica	Intragastric administration	Antiemetic effects	Inhibiting cisplatin-induced NLRP3 inflammasome activation Meng et al. (2020).
*Chen pi*	*Citrus reticulata* Blanco	Compound	Nobiletin	CCI-induced neuropathic pain mice	Intragastric administration	Analgesic effects	Inhibiting the IRF5/P2X4R/BDNF signalling pathway in spinal microglia Zhu et al. (2022)
		Extract (CO_2_)	Polymethoflavones and terpenes	CUMS mice	Intragastric administration	Antidepressant effects	Decreasing the content of monoamine oxidase in the cerebral cortex Li et al. (2021b).
		Extract (CO_2_)	D-limonene	Rat	Intragastric administration	Sedative and Hypnotic effects	The citrus essential oil significantly decreased REM sleep latency and increased total time and episode numbers of REM sleep Kwangjai et al. (2021).
*Bai shao*	*Paeonia lactiflora* Pall.	Compound	Paeoniflorin	CCI-induced neuropathic pain mice	Intragastric administration	Analgesic effects	Decreasing the levels of TNF-α and IL-1β proinflammatory cytokines in the spinal cord, inhibiting the over-activation of microglia and reducing the elevated expression levels of p-p38 MAPK and NF-κb in the spinal cord Zhou et al. (2017).
		Extract (water)	Tetradecane, Pentadecane, Myrtanal, Hexadecanoic acid, methyl ester, 2-Heptadecanone, Hexadecanoic acid, ethyl ester, Paeonol, Lauric acid, Methyl linoleate, Tetradecanoic acid, Dibutyl phthalate, Myristelaidic acid, Pentadecanoic acid, n-Hexadecanoic acid, Palmitoleic acid, Heptadecanoic acid, cis-10-Heptadecenoic acid, Stearic acid, Oleic acid, Linoleic acid, Linolenic acid	Corticosterone-induced depression mice	Intragastric administration, *In vitro*	Anti-apoptotic effects Antidepressant effect	Regulating PI3K/Akt/Nrf2 signalling pathway Sun et al. (2022).
*Xiang fu*	*Cyperus rotundus* L.	Extract (ethanol)	Phenols, tannins, glycoside, and flavonoids	Sodium nitrite-induced hypoxia rats	Intragastric administration	Neuroprotective effects	Cyperus rotundus possesses a protective effect against sodium nitrite-induced hypoxia in rats Jebasingh et al. (2014).
		Extract (ethanol)	Cyperi rhizome	CCI-induced neuropathic pain rat	Intragastric administration	Antinociceptive effects	Decreasing the nerve injury-induced mechanical allodynia, alleviating thermal heat hyperalgesia, reducing the nerve injury-induced phosphorylation of NMDA receptor NR1 subunit in the spinal dorsal horn Choi et al. (2012)
		Extract of *Chuanxiong* Rhizoma and Cyperi Rhizoma (1:2, ethanol)	Ferulic acid, senkyunolide A, 3-n-butylphthalide, Z-ligustilide, Z-3-butylidenephthalid, cyperotundone, nookatone and α-cyperone	Nitroglycerin-induced migraine rat	Intragastric administration	Anti-migraine effects	Increasing the cerebral blood flow, decreasing the expression of CGRP and c-fos mrna, and regulating the releasing of endothelin-1, GABA, NOS, 5-HT, 5-HIAA, CGRP and β-EP in the serum and brainstem Wu et al. (2019).

Note: 5-HT: 5-hydroxytryptamine, 5-HIAA: 5-hydoxyindoleacetic acid; BDNF: brain-derived neurotrophic factor; β-EP: β-endorphin; CCI: chronic constriction injury; CGRP: calcitonin gene-related peptide; CK-MB: creatine kinase-myocardial band; CUMS: chronic unpredictable mild stress; DA: dopamine; ET: endothelin; GABA_A_: γ-amino butyric acid type A; HMGB1: high mobility group box 1 protein; GABA: γ-aminobutyric acid; IL-1β: interleukin-1β; IL-6: interleukin-6; MAPK: mitogen-activated protein kinase; N/A: not applicable; NF-κB: Nuclear factor kappa-light-chain-enhancer of activated B cells; NLRP3: nucleotide binding and oligomerization domain-like receptor family pyrin domain-containing 3; NMDA: N-methyl-D-aspartate; NO: nitric oxide, NOS: nitric oxide synthase; NREM: nonrapid eye movement; REM: rapid eye movement; TNF-α: tumor necrosis factor-α. * Botanical names based on the World Flora Online (WFO) Plant List (https://wfoplantlist.org/accessed 31 October 2023).

The frequently used PCHMPs exhibit diverse functions, including modulating gastrointestinal functions, improving sleeping quality, relieving neck pain and headache, regulating emotions, and treating stroke, as instructed in the Chinese Pharmacopoeia 2020 (State Pharmacopoeia Committee of China, 2020). According to the Chinese medicine holistic theory, various organs and systems interact with each other. Since migraine is a neurological condition often comorbid with depression, anxiety and sleeping disorders, and commonly presents with gastrointestinal symptoms like vomiting and nausea, it is understandable that PCHMPs prescribed for migraine patients would aim to address these issues. Anti-migraine effects of some specific PCHMPs like *Tian shu* capsule and *Tong tian* oral solution have been confirmed in clinical trials (Xia et al., 2013; Yu et al., 2019; Liu, 2021a; Liu., 2021b; Lu, 2021). Specifically, *Tian shu* capsule/tablet achieved its analgesic effects via regulating calcitonin gene-related peptide, adenosine A2a receptor and adenosine A1 receptor (Lu et al., 2016). In addition, *Tian shu* capsule/tablet also exhibited anti-depression effects in mice model via regulating 5-hydroxytryptamine, dopamine, and norepinephrine in brain (Sun et al., 2018).

### 4.2 Implication for clinical practice and clinical research

#### 4.2.1 Implication for clinical practice

Presently, evidence supporting the use of CHM for migraine-comorbid depression and insomnia remains insufficient. However, there is practical merit in considering CHM as a recommended approach to mitigate migraine severity, alleviate anxiety symptoms, and enhance migraine patients’ quality of life. A minimum treatment duration of 28 days is suggested to achieve these effects based on the results of the current study. The finding may fill in the gap of CHM treatment duration for migraine. Unfortunately, patient adherence and persistence with treatment regimen are often lacking and inadequate, resulting in many patients receiving an insufficient duration of CHM therapy for their migraines.

It is crucial to emphasise the positive correlations between an extended treatment duration and the potential for enhanced treatment outcomes. Disseminating this information to migraine patients could serve to bolster their commitment to treatment adherence and persistence.

Within real-world clinical practice, CHM decoctions for migraines can be modified based on classical formulae, tailored to individual patent characteristics and symptoms. Additionally, prescribing PCHMPs guided by holistic principles and syndrome differentiation is a viable strategy.

The excessive use of acute medications is widely acknowledged as a risk factor for chronic migraine (Cevoli et al., 2009). Given CHM’s potential to alleviate migraine pain, it could serve as a valuable complementary approach alongside traditional acute medications. However, it is important to note that patients demonstrate suboptimal adherence to acute medication instructions, coupled with limited awareness of the necessity of reducing acute medications. While patient education has been shown to enhance clinical effectiveness for migraine treatment (Probyn et al., 2017), the availability of patient education remains inadequate, especially concerning chronic migraine (Short, 2019). As a result, incorporating patient education on appropriate acute medication usage and the perils of medication overuse in migraine management is imperative.

#### 4.2.2 Implication for clinical research

In the realm of clinical research for migraine, it is advisable to incorporate assessments of comorbid anxiety, depression, and insomnia into trial designs. This inclusion is vital for addressing the preferences and values of migraine patients. Moreover, when designing clinical trials to assess the effects of CHM on migraines, a minimum intervention period of 28 days is recommended, based on the GLMM analyses of this study. Additionally, it is advisable to conduct controlled clinical trials to investigate different durations of CHM treatment to further deepen our understanding of the relationship between the length of treatment and the therapeutic effects.

### 4.3 Limitations and generalisability

As a cohort study, several biases could originate from various stages throughout the study, including selection bias and confusion bias (Barría and Barría, 2018). However, effective measures have been implemented to control and minimise potential biases. Firstly, standardised inclusion criteria and a rigorous screening procedure were introduced to select representative migraine participants using a consecutive recruiting method. This method not only maintains external validity but also encompasses participants from a typical age range with a moderate disease duration, covering various migraine subtypes. Consequently, the study’s findings hold significant potential for sustainable generalisability. Secondly, strict follow-up plans with scheduled reminders and flexible response methods were employed to minimise the loss of follow-up bias. Thirdly, sophisticated multivariate analytical techniques were employed to minimise the confusion bias.

However, some inevitable limitations have been identified in this study. Firstly, the actual number of registered participants fell below the estimated count, and the rate of loss to follow-up remained noteworthy due to COVID-19-related quarantines, which may have influenced the availability of follow-up data. Secondly, due to its single-centre nature situation in southern China, the generalisability of the results might be compromised, limiting their applicability to migraine patients and Chinese medicine clinicians primarily in southern China. Moreover, the exceptional predominance of female participants in the study may restrict the generalisability of the findings to male migraine patients. This aspect warrants further investigation in future studies.

Nevertheless, it is important to recognise that the clinical expertise derived from real-world clinical practice, without researchers’ interference, adds practicality and relevance to clinical applications.

## 5 Conclusion

In real-world clinical practice, migraine patients undergoing an extended course of CHM (≥28 days) exhibited more severe migraine severity at baseline. However, they also achieved significant improvements in terms of monthly migraine days, peak pain NRS score, anxiety, and MSQ, which align with their primary preferences and values. Nonetheless, the current CHM treatment strategy employed in this study did not demonstrate effectiveness in addressing comorbid depression. Conversely, when administrated for less than 28 days, CHM treatment appeared to contribute primarily to the reduction of migraine pain and improvement in MSQ, without conferring sufficient benefits in preventing migraine attacks or addressing comorbidities.

## Data Availability

The original contributions presented in the study are included in the article/Supplementary material, further inquiries can be directed to the corresponding authors.

## Abbreviations

AE, adverse event; CHM, Chinese herbal medicine; CRF, case report form; GAD-7, generalised anxiety disorder 7-item; GLMM, generalised linear mixed model; GPHCM, Guangdong Provincial Hospital of Chinese Medicine; ICHD-3, International Classification of Headache Disorders, third edition; ISI, insomnia severity index; MSQ, migraine-specific quality of life questionnaire; MSQ-EF, migraine-specific quality of life questionnaire-emotional function; MSQ-RFP, migraine-specific quality of life questionnaire-role function-preventive; MSQ-RFR, migraine-specific quality of life questionnaire-role function-restrictive; NRS, numeric rating scale; NSAIDs, nonsteroidal anti-inflammatory drugs; PCHMP, patented Chinese herbal medicine product; PHQ-9, patient health questionnaire-9.
